# Selective prediction of interaction sites in protein structures with THEMATICS

**DOI:** 10.1186/1471-2105-8-119

**Published:** 2007-04-09

**Authors:** Ying Wei, Jaeju Ko, Leonel F Murga, Mary Jo Ondrechen

**Affiliations:** 1 Department of Chemistry and Chemical Biology and Institute for Complex Scientific Software, Northeastern University, Boston, Massachusetts 02115 USA; 2 NSF-ROA awardee. Department of Chemistry, Indiana University of Pennsylvania, 975 Oakland Avenue, Indiana, Pennsylvania 15705 USA; 3 Rosenstiel Basic Medical Sciences Center, Brandeis University, Waltham, MA 02454 USA

## Abstract

**Background:**

Methods are now available for the prediction of interaction sites in protein 3D structures. While many of these methods report high success rates for site prediction, often these predictions are not very selective and have low precision. Precision in site prediction is addressed using Theoretical Microscopic Titration Curves (THEMATICS), a simple computational method for the identification of active sites in enzymes. Recall and precision are measured and compared with other methods for the prediction of catalytic sites.

**Results:**

Using a test set of 169 enzymes from the original Catalytic Residue Dataset (CatRes) it is shown that THEMATICS can deliver precise, localised site predictions. Furthermore, adjustment of the cut-off criteria can improve the recall rates for catalytic residues with only a small sacrifice in precision. Recall rates for CatRes/CSA annotated catalytic residues are 41.1%, 50.4%, and 54.2% for Z score cut-off values of 1.00, 0.99, and 0.98, respectively. The corresponding precision rates are 19.4%, 17.9%, and 16.4%. The success rate for catalytic sites is higher, with correct or partially correct predictions for 77.5%, 85.8%, and 88.2% of the enzymes in the test set, corresponding to the same respective Z score cut-offs, if only the CatRes annotations are used as the reference set. Incorporation of additional literature annotations into the reference set gives total success rates of 89.9%, 92.9%, and 94.1%, again for corresponding cut-off values of 1.00, 0.99, and 0.98. False positive rates for a 75-protein test set are 1.95%, 2.60%, and 3.12% for Z score cut-offs of 1.00, 0.99, and 0.98, respectively.

**Conclusion:**

With a preferred cut-off value of 0.99, THEMATICS achieves a high success rate of interaction site prediction, about 86% correct or partially correct using CatRes/CSA annotations only and about 93% with an expanded reference set. Success rates for catalytic residue prediction are similar to those of other structure-based methods, but with substantially better precision and lower false positive rates. THEMATICS performs well across the spectrum of E.C. classes. The method requires only the structure of the query protein as input. THEMATICS predictions may be obtained via the web from structures in PDB format at:

## Background

Methods are now available for the prediction of interaction sites in protein 3D structures. While many of these methods report high success rates for site prediction, often these predictions are highly delocalised, span a significant fraction of the protein's surface, and are not very selective. Precision in the prediction of sites is addressed using THEMATICS, a simple computational method for the identification of enzyme active sites from the three-dimensional structure alone [1-7]. One goal of the present paper is to show how the already good performance of THEMATICS can be improved and to quantify the recall and precision rates of the method through comparison of its predictions with the information from a database of enzymes with experimentally identified active sites. The most important finding of the present work is that this simple, electrostatics-based method in fact delivers superior precision, *i.e. *well-localised site predictions with better selectivity than other structure-based methods, in addition to good recall. It is established here that THEMATICS applies to enzymes of many different structural and chemical classes. It is also demonstrated that the method works well for structures that do not contain a bound ligand, which is the situation for most structural genomics proteins and other proteins of unknown function. The identification of the interaction sites in protein structures is a critical step in the determination of function from the wealth of sequence and structure information emerging from genome sequencing and from structural genomics efforts [8-14].

Our method is based on established computational techniques and utilizes a finite difference Poisson-Boltzmann (FDPB) method [15-24] to calculate the Theoretical Microscopic Titration Curves – THEMATICS – for all of the ionisable residues in the protein. FDPB methods have been in use for two decades to calculate the pK_a_'s of ionisable residues in proteins. We have shown that the shapes of the theoretical titration curves generated from a FDPB method, although they are only approximate, contain a great deal of useful information about the location, binding properties and chemical properties of the active site [1,6,7].

A typical ionisable residue in a protein obeys the Henderson-Hasselbalch (H-H) equation, which is generally written as:

pH = pK_a _+ log{[A^-^]/[HA]}.

Equation (1) may be rewritten to express the mean net charge C (for a specified residue averaged over an ensemble of protein molecules) as a function of the pH, as:

C_+_(pH) = 10^pKa^/(10^pH ^+ 10^pKa^)

for the residues that form a cation upon protonation (Arg, His, Lys, and the N-terminus). These residues go from a +1 charged state to a 0 charge state as the pH is raised. Equation (2) is rewritten as:

C_-_(pH) = - 10^pH^/(10^pH ^+ 10^pKa^)

for the residues that form an anion upon deprotonation (Asp, Cys, Glu, Tyr, and the C-terminus). These residues go from a 0 charge state to a -1 charged state as the pH is raised. Equations (2) and (3) have the familiar sigmoid shape that is typical of a weak acid or base that obeys the H-H equation; as the pH is raised, these residues change from their protonated to deprotonated states in a narrow pH range. It is commonly (and not always correctly) assumed that when the pH is less than the pK_a_, the species is protonated and that when the pH is greater than the pK_a_, the species is deprotonated. While this is true for most of the ionisable residues in a protein, it has been reported previously [25-28] that a small number of residues have predicted titration curves with perturbed shapes that do not fit the H-H equation. We have demonstrated [1] that these perturbed curves are indeed significant because they occur in catalytic and binding sites with high frequency and with lower frequency elsewhere. In particular, ionisable residues involved in catalysis and/or recognition tend to have perturbed theoretical titration curves with flat or nearly flat regions in their predicted C(pH) functions. Therefore both protonated and deprotonated forms are significantly populated over a pH range that is significantly wider than that of the more typical residues. Recently Ko [6] reported on statistical metrics for the quantification of the deviations of a computed titration curve from H-H behaviour; the residues that deviate the most from H-H behaviour are then selected by statistical criteria. We have shown that these types of perturbed residues can be used to predict interaction sites, such that a cluster of two or more of these perturbed residues in three-dimensional space is a reliable predictor of active site or binding site location. Thus from the structure alone one can identify interaction sites, in the absence of further biochemical data, with just a simple and relatively fast calculation.

Most of the methods currently in use to predict the function of a protein from its sequence or from its structure rely on relationships to proteins of known function. For some classes of proteins, information about function can be inferred from the sequence [29-33]. However, these inferences can be misleading. Such methods also do not necessarily identify or characterize interaction sites. Analysis of sequence and structure data together gives more revealing clues about function [34-37]. Methods to locate active sites generally rely either on analogies to related proteins of known function [38-45], or on searches for clefts in the protein structure [46]. Energetics, flexibility and dynamics [47-50] may also serve as markers of function. The method of Gutteridge *et al. *[51] is based on sequence conservation and structural features and predicts active sites with a high success rate. This method returns a correct prediction for 69% of the 159 proteins in the test set and a partially correct prediction for 25% of the test proteins, with an average of 7.2 predicted clusters per protein.

Methods for the determination of functional information that utilize the structure of the query protein alone are relatively new. THEMATICS thus represents a departure from previous approaches because it takes advantage of the unique chemical and electrostatic properties of catalytically active sites in protein structures to identify and characterize them. Specifically, it searches for anomalies in the theoretical titration behaviour of the ionisable residues [1]. These anomalous titration curves arise from coupling between protonation events on the ionisable residues in the active site and on multiple ionisable partners. These couplings contribute most to titration curve anomalies when the electrostatic interaction is strong and when the pK_a_'s are roughly matched. While all ionisable residues in a protein experience such couplings, these couplings tend to be the strongest for active site residues. Structural Analysis of Residue Interaction Graphs (SARIG) [52] is another method based on interactions between residues, but it effectively counts all types of residue contacts, based on spatial proximity. It is a graph theoretic approach that calculates residue contacts and identifies the residues that have the highest closeness scores to all other residues. SARIG successfully predicts 46.5% of the annotated catalytic residues for the enzymes in the CatRes [53] database. The precision, however, is low; only 9.4% of the predicted residues are known catalytic residues. Still another computational approach to the identification of interaction sites from the structure alone involves solvent mapping. Originally this was developed as an experimental technique [54], but now entails the computational docking of small solvent molecules onto the protein surface and searching for clusters of energy minima for these molecules [55,56]. Q-SiteFinder, a simple and fast version of this method developed by Laurie and Jackson [57], uses only a methyl group as the probe. For 90% of proteins in the test set, Q-SiteFinder returns a correct prediction within the top three predicted sites, albeit with low precision. Another 3D-structure-based method based on purely geometric properties has been reported by Ben-Shimon and Eisenstein [58], for which a high success rate is reported for site prediction but performance data for catalytic and binding residue prediction is not reported.

While there are now methods available that predict catalytic residues from the 3D structure alone with good recall rates, it is desirable to select such residues with good precision, *i.e. *to obtain predictions where a higher fraction of the predicted residues are known catalytic residues. One of the goals of the present paper is to establish that catalytic sites and residues can be predicted using computed protonation properties with good recall and also substantially better precision. Precise, localised predictions are important for future applications, such as ligand design and also for comparative studies of predicted sites in proteins of unknown function with known sites in well-characterized proteins.

Having established the basic principles upon which THEMATICS works [1,2,4] and having developed a method to automate it [6], it is now possible and desirable to test it on a large set of enzymes that spans a wide range of chemical functions and structural types. In particular, we now apply THEMATICS to an annotated set of enzymes to measure the success rate and the precision and to study the degree of improvement obtainable in the recall without excessive loss of precision. We utilize the CatRes database [53], a compilation of information from the experimental literature that identifies residues in a protein structure that are involved in catalysis.

The method of reference [6] for the selection of those ionisable residues that deviate most from Henderson-Hasselbalch behaviour is based on a moment analysis. In particular the third and fourth moments of the derivatives of the FDPB-hybrid-computed titration curves are used to quantify deviation from H-H behaviour. In reference [6] a Z score is used to select those residues with third or fourth moments that are more than one standard deviation above the mean for all ionisable residues in the protein, *i.e. *Z_3 _> 1 or Z_4 _> 1. Here we examine how the Z score cut-off affects recall and precision and show how this cut-off may be adjusted to optimise the method for desired performance.

There are 178 proteins in the original CatRes database [53]. Nine of these have been excluded from the present analysis for specific reasons. One, Ribonuclease P [PDB: 1A6F], does not have any annotated catalytic residues and this is noted in the CatRes database. Four enzymes are excluded because of poor structure quality (*i.e. *a large number of missing atoms and/or residues in the structure file) or redundancy. Two others had to be excluded because the structures are too large for the current system to handle. Two enzymes are excluded from the present analysis because they are NMR structures. While THEMATICS can work for NMR structures, the analysis is different because there is a set of structures instead of a single structure. The present paper is based on the analysis of the x-ray crystal structures of 169 enzymes. The enzymes in the test set span a wide range of chemical functions. Table 1 shows the number and percentage of enzymes in the test population by EC class. For the 169 enzymes in our test set, a total of 594 residues are annotated as catalytically important in the CatRes database [53]. The CatRes information was checked against the Catalytic Site Atlas (CSA) [59,60] and for the applicable cases, the list of catalytically important residues was modified to incorporate the updated CSA annotations.

**Table 1:** Functional Class Distribution in the Test Set of 169 enzymes.

**Class**	**Number**	**Percent**
EC 1 Oxidoreductases	39	23%
EC 2 Transferases	39	23%
EC 3 Hydrolases	46	27%
EC 4 Lyases	28	17%
EC 5 Isomerases	9	5%
EC 6 Ligases	8	5%

In the present paper both the success rate for the prediction of sites and the success rate for the prediction of important residues are reported. We regard the labelled data sets as reliable sources of information about catalytic sites; *i.e. *the local area on or near the surface of a protein where the labelled residues are located is very likely to be a site where catalysis and/or binding occurs. Furthermore, the residues labelled as positive (catalytically important) have experimental evidence to support that labelling and therefore are considered reliably annotated. However, because the literature annotations of catalytic residues are incomplete, the absence of a positive annotation for a particular residue is not necessarily a reliable negative annotation. Therefore the computed precision rate for residues, the number of predicted residues that are annotated as important divided by the total number of residues predicted for a given protein, should be regarded as a lower bound.

To measure the performance of the method for catalytic residue prediction, three metrics are employed: Recall, Precision, and Filtration Ratio (FR). The recall (or sensitivity) is defined as the fraction of known active site residues that are predicted by the method, as:

Recall = (# of positive residues predicted)/(# of annotated positive residues)

Here a positive residue is one that is annotated in the reference database as an active site residue. The precision, related to the selectivity and to the specificity, is defined as the fraction of predicted residues that are known positives, as:

Precision = (# of positive residues predicted)/(total # of residues predicted)

Another measure of the selectivity is the filtration ratio (FR), the fraction of all residues that are predicted as positive, as:

FR = (# of residues predicted)/(total # of residues)

Thus the goal is to maximize recall and precision with low filtration ratio. The recall and precision for THEMATICS predictions are measured against the CatRes/CSA databases, so the annotations therein are used to determine the set of known positive residues used in equations (4) – (6). We recognize that the literature annotations are necessarily incomplete and are being updated continuously. In order to test performance, we designate the CatRes/CSA database as the best compiled reference set available for catalytic residue annotation.

The present study of the effect of Z score cut-off and the method comparison study therefore are performed using only the CatRes/CSA annotations. Thus the actual precisions are probably higher than the precisions calculated herein for all methods, since any of the methods may be predicting important residues that are not currently annotated as such in the database.

In a separate, subsequent analysis, some annotations from different sources are added to the CatRes/CSA information in an attempt to obtain a more realistic value for the performance metrics for sites. In particular, three different sets of reference annotations are used: Reference Set 1) is CatRes/CSA only; Reference Set 2) consists of Reference Set 1 plus PDB SITE entries and, in a few cases, additional literature articles; and Reference Set 3) is Reference Set 2 plus ligand-binding residues, as determined by the LPC [61] server, for cases where bound structures are available.

## Results

### Performance for residues as a function of Z cut-off for the 169-protein test set

First THEMATICS performance for residue prediction is measured as a function of the Z score cut-off. The metrics used in reference [6] are measures of deviation from H-H behaviour. For a Z score cut-off of 1.0, those residues with a metric that is more than one standard deviation above the mean value for all ionisable residues in a given protein are designated as positive. When the Z score cut-off is reduced, more residues are then predicted to be important.

**Figure 1:**
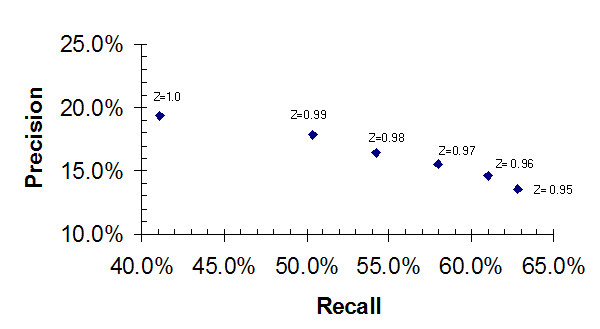
**Precision Versus Recall – THEMATICS predictions for CSA**. Precision (fraction of predicted residues that are known catalytic residues) as a function of recall (fraction of known catalytic residues that are predicted as positive) for THEMATICS predictions on the annotated set of 169 proteins. Known catalytic residues are defined here as only the CatRes/CSA annotated residues. Results are shown for Z score cut-off values of 1.0, 0.99, 0.98, 0.97, 0.96, and 0.95.

Table 2 shows the values for the recall and the precision obtained for the predicted active site residues, averaged over the 169 proteins in the test set, for Z score cut-off values ranging from 1.00 through 0.95. A Z score cut-off of 1.00, the value used by Ko [6], achieves a recall of CatRes/CSA annotated catalytic residues of 41.1% and a precision of 19.4%. When the Z score cut-off is reduced to 0.99, the recall increases to 50.4%; there is only a small concomitant drop in the precision, to a value of 17.9%. A Z score cut-off of 0.98 increases the recall to 54.2%, while the precision drops to 16.4%. Further reductions in the Z score cut-off return better sensitivities, up to 62.8% for a 0.95 cut-off, but always with some sacrifice in the precision. Figure 1 represents these data graphically, with precision plotted as a function of the recall for Z score cut-off values between 1.00 and 0.95.

**Table 2:** Recall and precision of CSA labelled residues.

**Z Score Cut-off**	**Recall**	**Precision**
Z = 1.00	41.1%	19.4%
Z = 0.99	50.4%	17.9%
Z = 0.98	54.2%	16.4%
Z = 0.97	58.0%	15.5%
Z = 0.96	61.0%	14.6%
Z = 0.95	62.8%	13.6%

Recall and Precision for THEMATICS predictions of catalytic residues as functions of Z score cut-off for the test set of 169 enzymes. Here only the CatRes/CSA annotations are used as the reference set. Values are averaged over the test set.

Thus a small reduction in the Z score cut-off, from 1.00 to 0.99 or to 0.98, leads to better recall with only a small reduction in precision. In particular, a Z score cut-off value of 0.99 yields a residue recall rate of better than 50% and at the same time gives a favourable precision rate of 17.9%.

While THEMATICS is able to predict roughly half of all known catalytic *residues*, the success rate for the prediction of catalytic *sites *is much higher.

### Overall performance for sites

First we compare the THEMATICS positive clusters against the CatRes/CSA annotations. The overwhelming majority of these enzymes have at least one ionisable residue labelled in the reference sets as catalytically active. Of the 169 enzymes in the test set, all but four contain one or more ionisable residue(s) annotated in the reference set as catalytically important. We therefore note that, if success is based solely on comparison with the specified reference labels, THEMATICS automatically fails for those four proteins, since THEMATICS in its present form only identifies ionisable residues. However, the percentage of proteins for which such automatic failure occurs is only 2.4%.

Following designations used in previous work [51], a site prediction is considered *correct *if it includes half or more of the annotated catalytic residues. A prediction is considered *partially correct *if it contains at least one, but less than half, of the annotated catalytic residues. The total success rate for the prediction of sites is the sum of the correct plus partially correct predictions. The success rates and the filtration ratio obtained for Z score cut-off values of 1.00, 0.99, and 0.98 are shown in Table 3 for the 169 CatRes enzymes. First our predictions are compared only with the CatRes/CSA annotations, which are the best available from a single source but incomplete. Then additional information about interaction sites from other reference sources is added for further comparison.

**Table 3:** THEMATICS success rates for site prediction.

**Z Score Cut-Off**	**Correct percentage based on CatRes**	**Partially Correct percentage based on CatRes**	**Total Success based on CatRes**	**Total % Success based on Reference Set 2**	**Total % Success based on Reference Set 3**	**Filtration Ratio**
1.00	48.5%	29.0%	77.5%	82.2%	89.9%	2.5%
0.99	59.8%	26.0%	85.8%	88.2%	92.9%	3.3%
0.98	66.9%	21.3%	88.2%	90.5%	94.1%	3.8%

Success rate is expressed as correct, partially correct, and total, using a Z score cut-off of 1.00, 0.99 and 0.98 for the test set of 169 enzymes. The total success rate is reported using three different reference sets of active site residues. Reference Set 1 is the CatRes/CSA database only; Reference Set 2 adds annotations from the PDB SITE field and associated journal articles; Reference Set 3 adds residues in contact with bound ligands.

Using only the CatRes/CSA lists of active site residues as the reference and using a Z score cut-off of 1.00, THEMATICS returns a correct prediction of the active site for 82 out of the 169 enzymes, a partially correct prediction for 49 enzymes, and an incorrect prediction for 38 enzymes. Thus the prediction is correct or partially correct for 131 out of 169 enzymes, or 77.5%, using Ko's [6] Z score cut-off value.

Further analysis was performed on the 38 enzymes where THEMATICS (with a Z score cut-off of 1.0) gave an incorrect prediction, according to the CatRes/CSA annotations. For these enzymes, the list of functionally important residues is augmented using additional information from the SITE field in the PDB file or from related journal articles. There are then eight additional successful predictions, for a total success rate of 82.2%, according to Reference Set 2. If we add the ligand binding residues computed by the Ligand-Protein Contacts (LPC) server [61] to the list of active site residues for those enzymes where a bound structure is available, the total success rate rises to 89.9%. These success rates are achieved with a low filtration ratio of 2.5%.

Improvement in the site prediction success rate is achieved with a slightly lower Z score cut-off, as shown in Table 3. Using a cut-off of 0.99, a correct site prediction is obtained for 101 enzymes and a partially correct prediction for 44 enzymes, for a total success rate of 85.8%, according to the CatRes/CSA data. This success rate increases to 88.2% against Reference Set 2 and to 92.9% against Reference Set 3, all with a filtration ratio of 3.3%.

If a Z score cut-off of 0.98 is used, then correct active sites are predicted for 113 of the enzymes and partially correct predictions for 36 enzymes, for a total success rate of 88.2%, using only the CatRes/CSA data as the reference. If Reference Set 2 is used as the source of true positive residues, then the overall success rate increases to 90.5%, and to 94.1% if Reference Set 3 is used. For a Z score cut-off of 0.98, the filtration ratio is 3.8%.

Based on these results and given our desire to make predictions with good sensitivity but without major sacrifice in precision, a Z score cut-off value of 0.99 is designated as the preferred value for our future predictive calculations.

### Comparison with other methods on a sample set

There are two other 3D-structure-based site prediction methods available for performance comparison, Q-SiteFinder [57] and SARIG [52]. It is only possible to compare performance on a subset of the CatRes/CSA database primarily because the online version of Q-SiteFinder is restricted to proteins with 10,000 atoms or fewer, because of the longer processing time for larger proteins. Thus a test set of 75 proteins, a subset of the CatRes/CSA set, was created such that the SARIG and Q-SiteFinder servers both return predictions for all members of the subset. Table 4 compares the performance of THEMATICS with these other 3D-structure-based methods on the test set of 75 proteins. The composition of the test set is described in the Methods section. THEMATICS performance is reported using Z score cut-off values of 1.00, 0.99, 0.98, 0.96, and 0.95. For purposes of Table 4, the combination of the top three sites is used as the Q-SiteFinder prediction; this combination of the top three sites was used in reference [57] as the basis for their calculation of the success rate. For the set of 75 proteins Reference Set 1 (only the CatRes/CSA annotations) is used for the labelled set; there are a total of 390 residues annotated therein as catalytic residues. Recall, precision, filtration ratio, and false positive fraction are calculated for the individual proteins and then averaged over the set of 75. The false positive fraction is defined as the number of predicted false positives over all negatives. Here a residue is taken to be negative if it is not annotated in the reference set as positive.

**Table 4:** Comparison of method performance for residue prediction on a sample set of 75 proteins.

**Method**	**Recall (%)**	**Precision (%)**	**Filtration ratio (%)**	**False Positive Percent**
THEMATICS Z = 1.0	48.1	25.0	2.5	1.95
THEMATICS Z = 0.99	53.4	21.9	3.2	2.60
THEMATICS Z = 0.98	56.5	19.5	3.8	3.12
THEMATICS Z = 0.96	65.6	17.0	4.8	4.08
THEMATICS Z = 0.95	67.5	16.1	5.3	4.54
Q-SiteFinder (top 3 sites)	65.6	5.4	14.9	14.3
SARIG	57.4	8.1	8.7	8.15

Recall, precision, and false positive rate are based on CatRes/CSA annotations and are averaged over the sample set of 75 proteins.

We note that the performance metrics for THEMATICS are a little better for this test set than on the entire CatRes dataset. Similarly, the recall rate for SARIG in the test set is 57%, better than the 46.5% reported in reference [52] for the entire CatRes database. We note that this smaller test sample is restricted to enzymes that return a result on the public servers for both SARIG and Q-SiteFinder and therefore this sample contains some inherent selection.

The three methods have a recall rate for known catalytic residues of 48–68% on the test set, with some variation among the methods within that range. Average recall rates are 66% for Q-SiteFinder and 57% for SARIG. THEMATICS recall rates are 48%, 53%, 57%, 66%, and 68% for Z score cut-offs of 1.00, 0.99, 0.98, 0.96, and 0.95, respectively. The primary difference in the performance of the three methods on the sample set is that THEMATICS has substantially higher precision. For THEMATICS with a Z score cut-off of 1.00, the average precision is 25%. The precision drops to 22%, 20%, 17%, and 16%, for Z score cut-offs of 0.99, 0.98, 0.96, and 0.95, respectively. Average precision rates for Q-SiteFinder and SARIG are 5% and 8%, respectively. Differences in mean precision between the methods are statistically significant. The 8% precision rate obtained for SARIG on the test set is close to the value of 9% reported in the original study [52] on the larger set. Since the database annotation is incomplete, the actual precision rates are probably higher for all of the methods, but values calculated using available annotations give some idea of the relative precision rates for the different methods. The low average filtration ratios in the 3–5% range obtained for THEMATICS predictions further demonstrate that on the average THEMATICS tends to yield more localised and less diluted predictions than the other two methods, for which higher filtration ratios were obtained. False positive fractions for THEMATICS are 1.95%, 2.60%, 3.12%, 4.08%, and 4.54% for the Z score cut-off values of 1.00, 0.99, 0.98, 0.96, and 0.95, respectively. The other methods show higher false positive rates: 14.3% for Q-SiteFinder and 8.15% for SARIG. For Z score cut-off values of 0.96 and 0.95, the THEMATICS residue recall rates are statistically equivalent to those of Q-SiteFinder, but the THEMATICS predictions show substantially better precision. SARIG is in the middle, with a precision rate between those of THEMATICS and Q-SiteFinder and a competitive residue recall rate.

**Figure 2:**
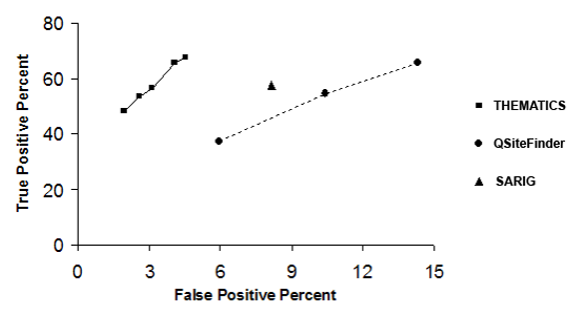
**Comparative ROC Diagram**. Comparative ROC diagram for THEMATICS (■), Q-SiteFinder (●), and SARIG (▲). True Positive Fraction (true positives predicted/all known positives) is plotted as a function of the False Positive Fraction (false positives predicted/all known negatives), both expressed as percentages, for a test set of 75 proteins. The CatRes/CSA annotations are used to designate the known positive residues and all other residues are taken to be negative. Five data points are shown for THEMATICS, corresponding to Z score cut-off values of 0.95, 0.96, 0.98, 0.99, and 1.00 (with ascending Z score values proceeding from upper right to lower left). The points for THEMATICS are joined by a solid line (—). The three points shown for Q-SiteFinder correspond to the top site only, a combination of the top two sites, and a combination of the top three sites (with ascending number of sites proceeding from lower left to upper right). The points for Q-SiteFinder are joined by a dashed line (- - - -).

Figure 2 compares the performance of THEMATICS (■), Q-SiteFinder (●), and SARIG (▲) in active site residue prediction with a Receiver Operating Characteristic (ROC) diagram. The True Positive Fraction (true positives predicted/all known positives), equivalent to the recall rate, is plotted as a function of the False Positive Fraction (false positives predicted/all known negatives) for the test set of 75 proteins. Both fractions are expressed as percentages. For the purposes of figure 2, the known positives are defined as the CatRes/CSA annotated residues and all other residues are assumed to be negative. Five data points are shown for THEMATICS, corresponding to Z score cut-off values of 0.95, 0.96, 0.98, 0.99, and 1.00, with ascending (*i.e. *increasingly selective but less sensitive) Z score values proceeding from upper right to lower left. The points for THEMATICS are connected by a solid line (—). Predictions for SARIG and Q-SiteFinder are based on results obtained from their respective public servers. Three points are shown for Q-SiteFinder and these correspond to the top site only, a combination of the top two sites, and a combination of the top three sites, with ascending number of sites (*i.e. *less selective but more sensitive) proceeding from lower left to upper right. Q-SiteFinder points are connected by a dashed line (- - - -).

### Performance across EC classes

Table 5 shows performance of THEMATICS on the 169 CatRes/CSA proteins by E.C. class. For purposes of Table 5, a 0.99 Z score cut-off is used. The success rates, recall, and precision are determined using only the CatRes/CSA annotations as the reference set. Filtration ratios by class range from 3.1% to 3.5%, clustering closely around the average value of 3.3% for the full set of proteins. Success rates and recall rates by class differ by about 10% or less of the respective average values for the full set. Precision rates vary a little more widely, although we note that the lowest values are obtained for E.C. classes 5 and 6; the values for these latter two classes should be regarded as approximate because of their small sample populations.

**Table 5:** THEMATICS performance on the CatRes/CSA proteins by E.C. class.

**E.C. Class**	**N**	**Success Rate**	**Recall**	**Precision**	**Filtration Ratio**
1	39	90%	55%	15%	3.1%
2	39	92%	54%	23%	3.3%
3	46	78%	45%	16%	3.4%
4	28	79%	50%	21%	3.5%
5	9	89%	45%	13%	3.2%
6	8	100%	48%	14%	3.4%
All	169	86%	50%	18%	3.3%

The Z score cut-off of 0.99 was used. Success rate, recall, and precision are based on CatRes/CSA annotations only.

### Structures with a bound ligand versus unbound structures

The CatRes/CSA database consists of a mixture of structures, some of which are not in complex (apo form) and others contain a bound small molecule (holo form). Applications of THEMATICS to proteins of unknown function, including most Structural Genomics proteins, requires that the method perform well for apo structures, because the natural substrate or ligand is nearly always absent and generally its identity is not even known. Of the 169 proteins in the test set, 72 of them contain no bound inhibitor or substrate-like molecule. Thus the performance of the method on this subset of unbound structures is compared with that of the full set. For purposes of these comparisons, a Z score cut-off of 0.99 is used and only the CatRes/CSA annotations are used as the reference set unless otherwise noted. For the 72 unbound structures, the average recall is 48.3%, compared to 50.4% for the full set of proteins. The average precision rate for the unbound subset is 19.1%, whereas 17.9% was obtained for the full set of proteins. The average filtration ratio was obtained as 3.3% for both the unbound subset and the full set. The overall success rate for the 72 unbound structures is 83% (60/72), based on CatRes/CSA annotations only; if additional annotations are added to the reference set, the success rate increases to 92% (66/72). These rates are close to the values obtained for the full set of 169 proteins: 86%, 88%, and 93%, for reference sets 1, 2, and 3 respectively (see Table 3).

THEMATICS performance on apo versus holo structures is further explored using pairs of structures in cases where both apo and holo structures are available for the same protein. Table 6 shows THEMATICS predictions for eight pairs of such structures. For each protein, results for the apo form are given in the first row and results for the holo form are given in the second row. The bound ligand and the PDB codes for each structure are also given. For each prediction, the residues that are in contact with the bound ligand, as determined with the holo structure and the LPC server [61], are shown in **boldface**. There are small differences in the predicted clusters between the two forms for most of these proteins. However, clusters containing correct ligand-binding residues are predicted for both the apo and holo structures for all eight proteins. For two of the eight proteins, the apo and holo structures yield identical predictions. For five of the eight proteins, the predicted clusters for the two forms contain the same set of residues in contact with the ligand, *i.e. *the set of residues in boldface is the same for the two forms. For β-amylase from *B. cereus*, both the apo and holo forms predict four correct ligand-binding residues, D49, H89, E172, and E367, but the holo structure yields one more – K287 – that is missed by the apo structure. However, there are also two examples in Table 6 where the apo form does a little bit better than the holo form. For the *S. typhi *ATP:corrinoid adenosyltranferase, both structures return three correct predictions, K41, E128, and Y131, but the apo form also correctly predicts R161. Likewise for retinol-binding protein II from rat, both the apo and holo structures predict Y19, but the apo form also correctly predicts Y60.

**Table 6:** THEMATICS predicted binding clusters for apo and holo structures.

**Protein**	**Ligand**	**Apo Holo PDB ID**	**THEMATICS Result**										
ATP:corrinoid adenosyltransferase	ATP	1G5R	**K41**	D127	**E128**	**Y131**	**R161**						
		1G5T	**K41**	D127	**E128**	**Y131**							
Intestinal Fatty Acid Binding Protein	MYR	1IFB	**Y14**	D34		**Y70**	**R106**	**Y117**	R126				
		1ICM	**Y14**	D34	R56	**Y70**	**R106**	**Y117**					
HPV11 regulatory protein	ALQ, 434	1R6K	**H29**	**H32**									
		1R6N	**H29**	**H32**									
Proline 3-hydroxylase	FE (II), sulfate	1E5R	**R97**	**H107**	H135	**H158**	R168	H238	Y239	H244			
		1E5S	**R97**	**H107**	H135	**H158**		H238	Y239	H244			
Glyoxalase 1	Ni(II)	1FA8	**H5**	**E56**	Y72	**H74**	D115	D117		**E122**			
		1F9Z	**H5**	**E56**	Y72	**H74**	D115		Y119	**E122**			
β-Amylase	maltose	1B90	Y14	Y44	**D49**	**H89**	Y164	**E172**	R174			Y310	**E367**
		1B9Z	Y14	Y44	**D49**	**H89**	Y164	**E172**	R174	Y178	**K287**	Y310	**E367**
GlcAT-P	UDP, Mn(II)	1V82	E101	R104	**D195**	**D196**	**D197**	Y200	D254				
		1V83	E101	R104	**D195**	**D196**	**D197**	Y200	D254				
Retinol-binding protein II	retinal	1OPA	**Y19**	**Y60**	C95		C121						
		1OPB	**Y19**		C95	R104	C121						

For each protein, the predicted binding cluster for the apo form is given in the top row and for the holo form in the second row. Predictions obtained using a Z score cut-off of 1.0. Residues that make contact with the ligand are shown in **bold**.

### Examples

**Figure 3:**
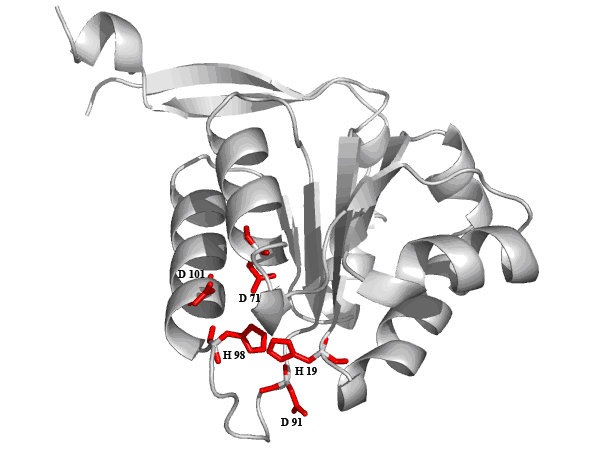
**THEMATICS predictions for Methylglyoxal synthase**. Ribbon diagram of one of the subunits of Methylglyoxal synthase (PDB: 1B93) with the side chains of the THEMATICS predicted catalytic residues H19, D71, D91, H98, and D101 shown explicitly in red; all of these are correctly predicted. Prediction was made using a Z score cut-off value of 0.99 and a 9 Å distance cutoff.

Some examples illustrate the localised nature of THEMATICS predictions. Figure 3 shows a ribbon diagram of one of the subunits of Methylglyoxal synthase (E.C. 4.2.3.3; PDB: 1B93) from *E. coli *with the side chains of the THEMATICS predicted residues shown explicitly in red. This prediction, obtained using the preferred Z score cut-off value of 0.99, is a five-member positive cluster consisting of H19, D71, D91, H98, and D101. The Catalytic Site Atlas [60] lists H19, G66, D71, D91, H98, and D101 as the important residues. H19, D71, and H98 are the known catalytic residues, while D91 and D101 are involved in substrate recognition [62-65]. The backbone amide group of G66 is also involved in substrate binding and is the only listed active residue that THEMATICS does not predict. The largest pocket for this structure, as computed by Computed Atlas of Surface Topography of Proteins (CASTp) [66,67], consists of a total of 20 residues and includes two of the six annotated residues, H19 and D91. The correctly predicted catalytic site overlaps with one corner of this largest pocket.

**Figure 4:**
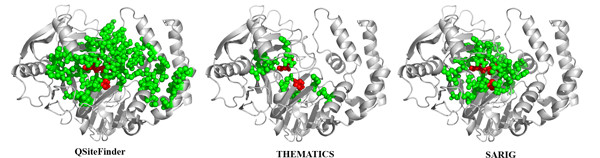
**Comparison of active site predictions for Adenylsuccinate synthase**. Active sites as predicted by Q-SiteFinder, THEMATICS, and SARIG for Adenylosuccinate synthase (E.C. 6.3.4.4; PDB: 1GIM) from *E. coli*. Atoms of predicted residues are shown as colored balls. Predicted residues annotated as correct according to CatRes/CSA are shown in red; other predicted residues are shown in green. The prediction shown for Q-SiteFinder consists of the top three sites. THEMATICS predictions were obtained using a Z score cut-off of 0.99 and a 9 Å distance cutoff.

Figure 4 shows three predictions for Adenylsuccinate synthase (E.C. 6.3.4.4; PDB: 1GIM) from *E. coli *obtained from Q-SiteFinder [57], THEMATICS, and SARIG [52]. The atoms of the predicted residues are shown as colored balls on the ribbon diagrams. Predicted residues listed in CatRes as catalytic residues, D13, H41, and Q224, are shown in red; other predicted residues are shown in green. The prediction shown for Q-SiteFinder consists of the top three sites. The THEMATICS predictions were obtained using the preferred Z score cut-off of 0.99 and a 9 Å distance cutoff. The THEMATICS predicted cluster consists of a total of ten residues: [D13, K16, H41, H53, E82, E221, K267, Y269, R305, K331] and includes D13 and H41, two of the three CatRes-listed catalytic residues. Q-SiteFinder and SARIG both predict all three listed catalytic residues, but Q-SiteFinder predicts a total of 38 residues and SARIG predicts a total of 32 residues; these predictions extend over a larger region than the THEMATICS prediction, as is apparent in Figure 4. For this particular example, there are five common residues predicted by the three methods: D13, K16, H41, R305, and K331. All five of these residues are either listed catalytic residues or ligand recognition residues.

**Figure 5:**
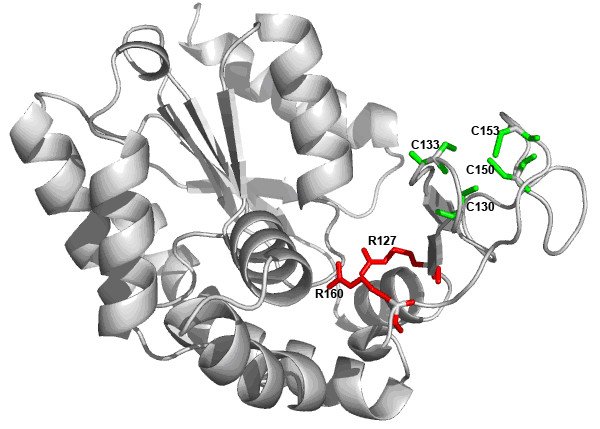
**THEMATICS predictions for Adenylate kinase**. Ribbon diagram showing THEMATICS predictions for Adenylate kinase from *B. stearothermophilus*. (PDB: 1ZIO). Side chains of predicted residues are shown explicitly in colour. Prediction was made using a Z score cut-off of 0.99 and a 9 Å distance cutoff. The side chains of the two residues R127 and R160 in the correctly predicted catalytic cluster are shown in red. An additional predicted cluster, a zinc-binding site, is shown in green.

Figure 5 shows THEMATICS predictions for Adenylate kinase (E.C. 2.7.4.3; PDB: 1ZIO) from *Bacillus stearothermophilus*. This is a case where THEMATICS does not predict the correct catalytic site using a Z score cut-off of 1.0 but the slightly lower, preferred cut-off value of 0.99 does return a prediction at the correct catalytic site. Using a Z score cut-off of 0.99, THEMATICS predicts the catalytic site [R127, R160] (shown in red in Figure 5) and an additional zinc-binding site [C130, C133, C150, C153], shown in green. This is because the cysteine residues in the second cluster [C130, C133, C150, C153], a cluster that coordinates a Zn^2+ ^ion that is structural in nature [68], exhibit very strong predicted interaction between their ionisation events. This can cause residues in the active site with anomalous titration behaviour to fall below the cut-off. The slightly lower Z score cut-off of 0.99 places two additional residues above the cut-off, so that a predicted cluster is formed around the catalytic residues R127 and R160. If the Z score cut-off is dropped further to 0.98, the predicted catalytic cluster [R36, R127, R160, D162, R171] includes two more catalytic residues, D162 and R171.

**Figure 6:**
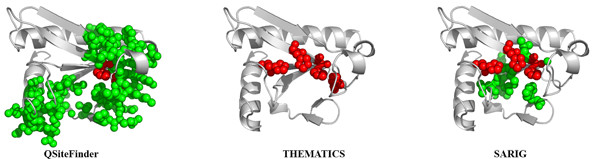
**Comparison of active site predictions for fragile histidine triad protein**. Active sites as predicted by Q-SiteFinder, THEMATICS, and SARIG for human fragile histidine triad protein (E.C. 3.6.1.29; PDB: 5FIT). Predicted residues known to be correct are shown in red; other predicted residues are shown in green. The prediction shown for Q-SiteFinder consists of the top three sites. THEMATICS predictions were obtained using a Z score cut-off of 0.99 and a 9 Å distance cutoff.

Figure 6 shows predictions by three methods for human Fragile Histidine Triad protein, FHIT (E.C. 3.6.1.29; PDB: 5FIT). Predictions are obtained from Q-SiteFinder (using the top three sites), THEMATICS (using a Z score cut-off of 0.99), and SARIG. The atoms of correctly predicted residues [69,70] are shown as red balls. Other predicted residues are shown as green balls. Again, THEMATICS returns a highly localised prediction.

### Predictions for Structural Genomics proteins

**Figure 7:**
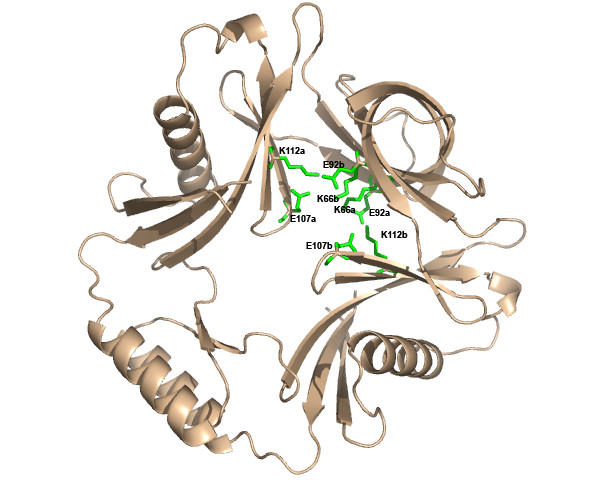
**THEMATICS predictions for novel fold and orphan sequence protein TM0875**. Ribbon diagram of TM0875 from *Thermatoga maritima *(PDB: 1O22), a structural genomics protein, showing the THEMATICS predictions. TM0875 is a hypothetical protein with a novel fold and an orphan sequence. The side chains of the residues in the THEMATICS predicted site are shown as green sticks and consist of the residues: [K66a, E92a, E107a, K112a, K66b, E92b, E107b, K112b].

**Figure 8:**
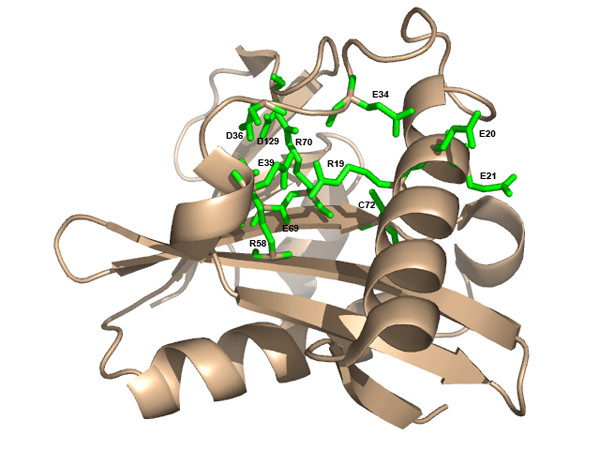
**THEMATICS predictions for structural genomics protein YJCF**. Ribbon diagram of structural genomics protein YJCF from *Bacillus subtilis *(PDB: 1Q2Y), showing the THEMATICS predictions. The side chains of the residues in the THEMATICS predicted site are shown as green sticks and consist of the residues: [R19, E20, E21, E34, D36, E39, R58, E69, R70, C72, D129].

The utility of THEMATICS for site prediction for structural genomics proteins, including novel folds and orphan sequences, is now illustrated with some examples. Figure 7 shows the THEMATICS prediction for the structural genomics protein TM0875 from *Thermatoga maritima*, (PDB: 1O22), a hypothetical protein with a novel fold and an orphan sequence. The side chains of the residues in the THEMATICS predicted site are shown as green sticks and consist of the residues [K66A, E92A, E107A, K112A, K66B, E92B, E107B, K112B]. Figure 8 shows the THEMATICS prediction for the YJCF protein from *Bacillus subtilis *(PDB: 1Q2Y), a structural genomics protein and a member of the GCN5-related N-acetyltransferase superfamily fold. The side chains of the residues in the THEMATICS predicted site are shown as green sticks and consist of the residues [R19, E20, E21, E34, D36, E39, R58, E69, R70, C72, D129].

## Discussion

Evaluation of the recall and precision rates for the entire set of 169 enzymes, as shown in Table 2, suggests that performance can be improved with a small reduction in the Z score cut-off to 0.99 or 0.98 from the 1.00 value used previously [6]. There is a significant increase in the recall with only a small sacrifice in precision. The good precision of the present method is one of its main advantages when compared with other available 3D-structure-based methods. Even with the less selective Z score cut-off values of 0.96 and 0.95, THEMATICS precision rates are still better than the other methods tested while recall rates are competitive. Further reduction of the Z score cut-off to values less than 0.95 does give further increase in the recall, but the precision starts to decrease to values approaching those of other methods and thus the selectivity advantage of THEMATICS wanes. Based on the results shown in Tables 2 and 3 and Figure 1, we adopt a Z score cut-off of 0.99 as the "preferred" value, in that it gives a high rate of successful site prediction (93%) while still maintaining our desired high precision rate. These data confirm what THEMATICS users have already observed empirically.

THEMATICS requires only the 3D structure of the query protein as input and therefore the query protein does not have to have any sequence homologues or similar structures and thus is applicable to a wider set of proteins than methods that are based on sequence homology. However, it is noted that THEMATICS performance does compare quite favourably with methods that do require sequence alignments. For instance, one recently reported site prediction method, based on sequence alignments and the 3D structure, reports a catalytic residue recall rate of 47% and a 5% false positive rate [71]. This constitutes better performance than some earlier methods based on sequence homology and 3D structure. Using only 3D-structure-based information, THEMATICS with a Z score cut-off of 0.99 does roughly as well in the recall rate (53%) but with a lower false positive rate of 2.6% on the 75-protein test set.

The present method of selection is based on perturbations in titration curve shape. This is quite different from selection based on electrostatic interaction energy or shift in pK_a_. Residues with anomalously shaped titration curves are few in number and tend to be localised in the active site. Residues with shifted pK_a_'s are greater in number and are more widely spread across the protein structure. An earlier study [47] showing that electrostatics- and titration- based methods give a large number of false positives for a 20-enzyme test set was based on a method of the latter type and thus is considerably less selective and less precise than the present method.

For residues that are predicted but not listed in the database as important, it is not clear at this time how many of them actually play a functional role and how many are simply false positives. Experiments are currently in progress to address this question.

Performance metrics for unbound (apo) structures appear to be about as good as those for bound (holo) structures. Predicted clusters for apo and holo forms are similar but not identical. FDPB methods are 3D-structure dependent and the predicted titration curves change as 3D structure changes. However, the strong electrostatic interaction between ionising events for the active residues is preserved sufficiently in the apo structures such that the statistical analysis can still identify them. Such capability for unbound structures is particularly important for the prediction of sites in proteins of unknown function. The pairs of apo-holo structures featured herein undergo changes primarily in side chain orientation upon ligand binding, *i.e. *ligand binding is accompanied by small changes in backbone conformation. Application to systems undergoing large changes in backbone conformation upon ligand binding, including allosteric systems, involves a number of additional issues beyond the scope of the present paper and is a subject of further exploration.

## Conclusion

Herein it has been established that our electrostatics-based method can actually predict sites from the 3D structure with better precision, lower filtration ratio, and lower false positive rate than other methods. THEMATICS works well on a diverse set of enzymes spanning all six EC classes, with similar performance data observed within each of the six EC classes.

It is also noted that the present method is successful with only one type of computed property used as input, namely the proton binding properties. The present results seem to point to the possibility that a combination of 3D structure based properties can lead to even better performance. SARIG and Q-SiteFinder have one obvious advantage over THEMATICS in that they can predict non-ionisable residues. It may be advantageous to combine capabilities.

THEMATICS predictions tend to be precise and well localised and thus may be suitable for applications such as ligand design or functional annotation based on comparison of predicted active site motifs.

## Methods

THEMATICS computations generally are performed on the biological unit for each enzyme in the dataset. Protein structures were obtained from the Protein Data Bank [72]. If there are missing side chain atoms, the Swiss PDB Viewer (SPDBV) [73,74] program is used to rebuild the missing atoms. The hydrogen atoms are built into the structure using TINKER [75] and the OPLS-UA force field [76,77]. Substrates, cofactors, water molecules, and ions that crystallize with the proteins are not included in the electric field calculations. The values for the dielectric constants are assumed to be 20 for the protein interior and 80 for the solvent. The theoretical titration curve for each ionisable residue is obtained using a Finite Difference Poisson-Boltzmann procedure. The University of Houston Brownian Dynamics program [19] (UHBD) is used to obtain the electrical potential function. The program HYBRID [17], calculates the average charge C as a function of pH using a hybrid Monte Carlo procedure. The first derivative (f) functions are obtained numerically by the 4-point formula and the moments of the f functions are calculated using Gaussian quadrature [78]. The Z scores Z_3 _and Z_4 _are calculated for each ionisable residue using the corresponding mean and standard deviation obtained for all ionisable residues in the same protein structure. Z score cut-offs evaluated in the present study run from 1.00 to 0.95. THEMATICS positive residues are defined as those residues with either Z_3 _or Z_4 _greater than the cut-off value Z_cut-off_, *i.e. *Z_3 _> Z_cut-off _OR Z_4 _> Z_cut-off_. THEMATICS positive residues are then assigned to clusters, where a residue is a cluster member if it is within 9 Å of another cluster member. For purposes of cluster definition, distances between residues are measured between the side chain atoms where the charge is centred in the ionised form of each residue. Clusters containing two or more residues are considered predictive. Single member clusters are reported but are not considered predictive.

For THEMATICS with Z score cut-off of 0.95 (the cut-off with the lowest precision obtained by THEMATICS on the 75-protein subset), the student T test with 95% confidence interval shows that the mean difference in precision between THEMATICS and SARIG, and between THEMATICS and Q-SiteFinder, is statistically significant. In other words, THEMATICS with Z score cut-off of 0.95 has higher precision than Q-SiteFinder and SARIG, and at the same time has similar or better recall value, as Table 4 shows. Differences in the mean values of performance metrics for THEMATICS on the six E.C. classes, as shown in Table 5, were found to be insignificant by a one factor ANOVA test with 95% confidence interval. The student T and ANOVA tests were performed with SPSS software.

To obtain the test set of 75 proteins for method comparison purposes, about half of the proteins within each of the six E.C. classes were chosen at random from the CatRes database to create a preliminary list. Proteins were then deleted from the list if either the Q-SiteFinder [79] or SARIG [80] servers were unable to return a prediction. The resulting test set of 75 proteins is a representative cross-section of the six E.C. classes.

A free THEMATICS web server at  is available to the public. The user submits the PDB ID or uploads the structure in PDB format. THEMATICS predictions are returned to the user by e-mail.

## Abbreviations

CASTp Computed Atlas of Surface Topography of Proteins

CatRes Catalytic Residue Database

CSA Catalytic Site Atlas

EC Enzyme Class

FDPB Finite Difference Poisson Boltzmann

FPF False Positive Fraction

FR Filtration Ratio

LPC Ligand-Protein Contacts

NMR Nuclear Magnetic Resonance

OPLS-UA Optimised Potential for Liquid Simulations – United Atom

PDB Protein Data Bank

ROC Receiver Operating Characteristic

SARIG Structural Analysis of Residue Interaction Graphs

SPDBV Swiss PDB Viewer

THEMATICS Theoretical Microscopic Titration Curves

UHBD University of Houston Brownian Dynamics

## Authors' contributions

YW performed the calculations and the data analysis and made the largest contribution to the interpretation of the results. All four authors contributed to the formulation of the problem, to the design of the methods of implementation, to the interpretation of the results, and to the preparation of the manuscript.
